# Urinary exosomal hsa_circ_0001250 as a novel diagnostic biomarker of idiopathic membranous nephropathy

**DOI:** 10.1186/s12967-022-03784-y

**Published:** 2022-12-19

**Authors:** Qianyu Li, Mingzhu Xu, Zhiping Zhang, Min Yin, Yucheng Zhang, Feng Liu

**Affiliations:** 1 Department of Nephrology, China-Japan Union Hospital of Jilin University, Changchun, Jilin China; 2 Scientific Research Center, China-Japan Union Hospital of Jilin University, Changchun, Jilin China

**Keywords:** Idiopathic membranous nephropathy, Exosomes, Circular RNAs, Bioinformatics, Biomarkers

## Abstract

**Aims:**

Idiopathic membranous nephropathy (IMN) is a common cause of adult nephrotic syndrome. Currently, the diagnosis of IMN mainly depends on renal biopsy, which is invasive. What’s more, markers already known for the clinical diagnosis of IMN are not sensitive enough. The present study aims to investigate the profiling of urinary exosomal circular RNAs (circRNAs) of IMN, and to look for a potential biomarker for diagnosis of IMN.

**Methods:**

Urine exosomes were collected from patients with IMN and idiopathic nephrotic syndrome (INS), as well as healthy controls (HCs) by ultracentrifuge. A pairwise comparison between 5 IMN and 5 HC was performed by high-throughput sequencing. Enrichment analysis were performed to explore the potential functions of differentially expressed circRNAs in IMN. Among three differentially expressed circRNAs which may be involved in signaling pathways of pathogenesis of IMN and matched conserved mouse circRNAs, hsa_circ_0001250 was selected as the target circRNA after quantitative polymerase chain reaction among 23 IMN, 19 INS and 23HC. Sanger sequencing and RNase R digestion assay were performed to validated the ring-structure and sequence of hsa_circ_0001250. ROC (Receiver Operating Characteristic) curve correlation analysis was used to further validate the potential utility of hsa_circ_0001250 as a diagnostic biomarker of IMN. A circRNA-miRNA-mRNA network was constructed to reflect the relationship between hsa_circ_0001250 and its target miRNAs and mRNAs.

**Results:**

766 up-regulated and 283 down-regulated circRNAs were identified in IMN patients. Kyoto Encyclopedia of Genes and Genomes pathway analysis revealed signaling pathways of pathogenesis of IMN which the different expressed circRNAs may participate in. The ring-structure and the sequence of hsa_circ_0001250 were confirmed, the expression of hsa_circ_0001250 was validated significantly increased in IMN, relevant with high level of proteinuria. A circRNA-miRNA-mRNA network reflected that hsa_circ_0001250 may play a role in the pathogenesis of IMN by target hsa-miR-639 and hsa-miR-4449.

**Conclusion:**

We revealed the expression and functional profile of differentially expressed urinary exosomal circRNAs of IMN patients. Urinary exosomal hsa_circ_0001250 was tested as a potential biomarker of IMN and a predicted circRNA-miRNA-mRNA network was constructed.

## Introduction

Membranous nephropathy (MN), which is characterized by the extensively thickened glomerular basement membrane (GBM), is a common cause of adult nephrotic syndrome, accounting for 20–30% of glomerular disease cases and some cases inevitably progressed to end-stage renal disease [1, 2]. MN can be classified as idiopathic membranous nephropathy (IMN) and secondary MN. It is widely accepted that IMN is an autoimmune disease mediated by the accumulation of antigen–antibody complexes outside the glomerular basement membrane, which leads to the activation of complement system and the changes in the podocyte morphology, causing proteinuria and further damage to the kidney [3]. However, the specific molecular pathogenesis is unclear. At present, the diagnosis of IMN mainly depends on renal biopsy [4], which is invasive. What’s more, there is still a lack of targeted treatment options. Therefore, the most urgent needs are to find non-invasive diagnosis markers and specific therapeutic targets of IMN.

Recently, researchers have studied the diagnostic capabilities of non-coding RNA in exosomes [5, 6]. Exosomes are extracellular vesicles with a diameter of 40–160 nm (average ~ 100 nm) originating from the endosomal pathway by a process including endocytosis, merging and release. Thus, many constituents of a cell, including DNAs, RNAs, lipids, metabolites, and cytosolic and cell-surface proteins can be contained [7]. The double-layer membrane structure of exosomes can protect the substance, so exosomes can be good carrier of molecular markers. Limited by the detection technology, it is only in recent years that researchers gradually recognize what exosome really is. Studies suggest that exosomes play a regulatory role that selectively encapsulate specific RNA molecules, deliver them to nearby or distant target cells, which is an important way of intercellular communication, effectively regulate the biological functions of target cells and closely related to the physiological and pathological processes of many diseases [8]. Exosomes were detected in a variety of body fluids [9], among which urinary exosomes have been proved to be a kind of marker of many kidney diseases, such as chronic kidney disease [10],diabetic kidney disease [11, 12], autosomal dominant polycystic kidney disease [13], clear cell renal cell carcinoma [14], renal fibrosis [15, 16], etc.

Among these non-coding RNAs, circular RNAs (circRNAs), different from traditional linear RNAs, are identified by the special covalently closed loop structure, for which they are more resistance to RNase R and thus have a longer half-life [17, 18]. A large number of studies have shown that circRNAs can be potential diagnostic marker and therapeutic target for they have a unique expression profile and important biological functions in a variety of diseases, such as tumor [19], cardiovascular disease [20], neurological disease [21] and autoimmune disease [22]. Studies have found that circRNAs can enter the extracellular space through exosomes, which suggests that they may be signal molecule for cell communication, and also shows the potentiality as a diagnostic marker [9].

Compared with other body fluids, urine, which is produced by kidney, seems to convey more information about kidney damage and diseases of the urinary system. Thus, the present study aims to look for a target urinary exosomal circRNA as a potential biomarker for diagnosis of IMN patients. In this study, we investigated the difference expression profile of circRNAs in urinary exosomes between patients with IMN and healthy controls by high-throughput sequencing and bioinformatics analysis. And we finally found that hsa_circ_0001250 have relevance in IMN and may be the potential biomarker for IMN.

## Materials and methods

### Clinical specimens and collection

A total of 47 age- and sex-matched patients with IMN and INS treated at China-Japan Union Hospital of Jilin University and 28 HCs were enrolled in this study. Among which, urinary samples from 5 IMN patients and 5 HCs were collected for RNA high throughput sequencing and bioinformatics analysis, then 23 patients with IMN, 23 HCs and 19 patients with INS as disease controls were enrolled for confirming the RNA sequencing results by qPCR assay and subsequent statistical analysis. Inclusive criteria of IMN or INS group were: diagnosed with IMN or INS by renal biopsy, didn’t receive any immunosuppressive treatment. Exclusive criteria were: patients with other diseases which can cause renal damage such as autoimmune disease, diabetes, hypertension or hepatitis B, or patients suffered diseases with aberrant circRNA expression such as infection, tumor, cardiovascular disease, neurological disease. Samples of blood and urine from these participants were collected at the nephrology department before renal biopsy. Their second-morning urine (approximately 100 ml) were collected into a sterilized centrifuge tube and then stored at -80℃. This study was approved by the Research Ethics Committee of China-Japan Union Hospital of Jilin University (No.2021-KYLL-060002). Written informed consent was obtained from all participants.

### Isolation of urinary exosomes

The urine samples were quickly melted at 37 °C in a water-bath and then centrifuged for 30 min at 2000 g, 4 °C. Afterwards, each tube of supernatant was carefully transferred to a new centrifuge tube and centrifuged for 45 min at 10,000*g*, 4 °C to remove larger vesicles. The supernatant was then filtered through a 0.45 μm filter and the filtrate were collected and then ultracentrifuged at 100,000*g* for 70 min at 4 °C. The sediment was resuspend with 10 mL pre-cooled 1 × PBS and were ultracentrifuged again at 4 °C, 100,000 g for 70 min. The sediment was resuspended with 100 μL of pre-chilled 1 × PBS, from which we took 5 μL for electron microscope, 50 μL for protein extraction, and 20 μL for RNA extraction.

### Detection of exosomes

#### Transmission electron microscopy (TEM)

In order to observe the morphology of exosomes, isolated exosomes were diluted with PBS buffer and then dropped onto a carbon-coated formvar grids for 1 min and stained with phosphotungstic acid for 1 min. Afterwards, the grid was dried at room temperature for several minutes and subsequently visualized on a transmission electron microscope (TEM; Hitachi, HT7700) at 100 kV.

#### Western blot assay

Western blot (WB) was used to test the specific exosomal markers as protein CD9, CD63, and calnexin was detected as a negative control. The whole proteins were lysed by RIPA Lysis Buffer. The protein concentration was measured by BCA method. After purified and electrophoretically separated, proteins were transferred to the polyvinylidene fluoride (PVDF) membrane. The membranes were blocked by 5% nonfat milk for 1 h and then incubated overnight at 4 °C with each primary antibodies, and subsequently incubated with HPR-labeled secondary antibodies at room temperature for 1 h. Finally, the enhanced chemiluminescence system (CLINX) was applied for signal visualization.

### Total RNA extraction and quality control

Total RNA in the urine was extracted using TRIzol reagent. The concentrations of the RNA were measured using a NanoDrop ND-1000 (Thermo Fisher Scientific, Waltham, MA, USA), the ratio OD260/OD280 is used to estimate the purity of obtained RNAs. OD260/280 ratio around 1.8–1.9 showed the high purity of the RNAs, demonstrated the good integrity of the obtained RNAs that could be used for later experiments.

### RNA library preparation, sequencing of circRNAs and bioinformatics analysis

RNA high throughput sequencing was performed by Cloud-Seq Biotech (Shanghai, China). The rRNAs were removed from total RNA with NEBNext rRNA Depletion Kit (New England Biolabs, Inc., Massachusetts, USA). After constructed by NEBNext® Ultra™ II Directional RNA Library Prep Kit (New England Biolabs, Inc., Massachusetts, USA), the RNA libraries were quality controlled and quantified with the BioAnalyzer 2100 system (Agilent Technologies, Inc., USA). Then library sequencing was performed on an illumina Hiseq instrument with 150 bp paired end reads harvested from Illumina HiSeq 4000 sequencer, and were quality controlled by Q30. The cutadapt [23] software (v1.9.3) was used to remove 3’ adaptor-trimming and low quality reads. The high quality trimmed reads were aligned to the reference genome/transcriptome with STAR software [24] (v2.5.1b) and circRNAs were detected and identified with DCC [25] software (v0.4.4). The identified circRNAs were annotated with circBase database [26] and Circ2Traits [27]. Then edgeR software [28] (v3.16.5) was used to normalize the data and perform differentially expressed circRNA analysis. The edgeR software was used to calculate log2FC value and *P* value of IMN and HC groups for the volcano plot, logCPM for the scatter plot, which were drawed with Python language and matplotlib package. Kyoto Encyclopedia of Genes and Genomes (KEGG) pathway (https://www.genome.jp/kegg/) is a database resource for understanding high-level functions and utilities of the biological system [29]. After the different expressed circRNAs were obtained, the Fisher’s test was performed for enrichment analysis according to the annotation of the KEGG pathway of each gene, screened the pathways with a *P* value < 0.05. The obtained results are drawn using the ggplot2 package for R. A map of ten miRNAs and their predicted target mRNAs was constructed of underlying molecular mechanisms through specific base pairing to demonstrate the circRNA-miRNA-mRNA network of hsa_circ_0001250 based on TargetScan (v7.0) [30] and miRanda (v3.3a) [31] software. The significant pairs in network were constructed using Cytoscape software (v3.1.0) [32].

### Validation of expression of circRNAs using quantitative reverse transcription PCR (RT-qPCR)

**Fig. 1:**
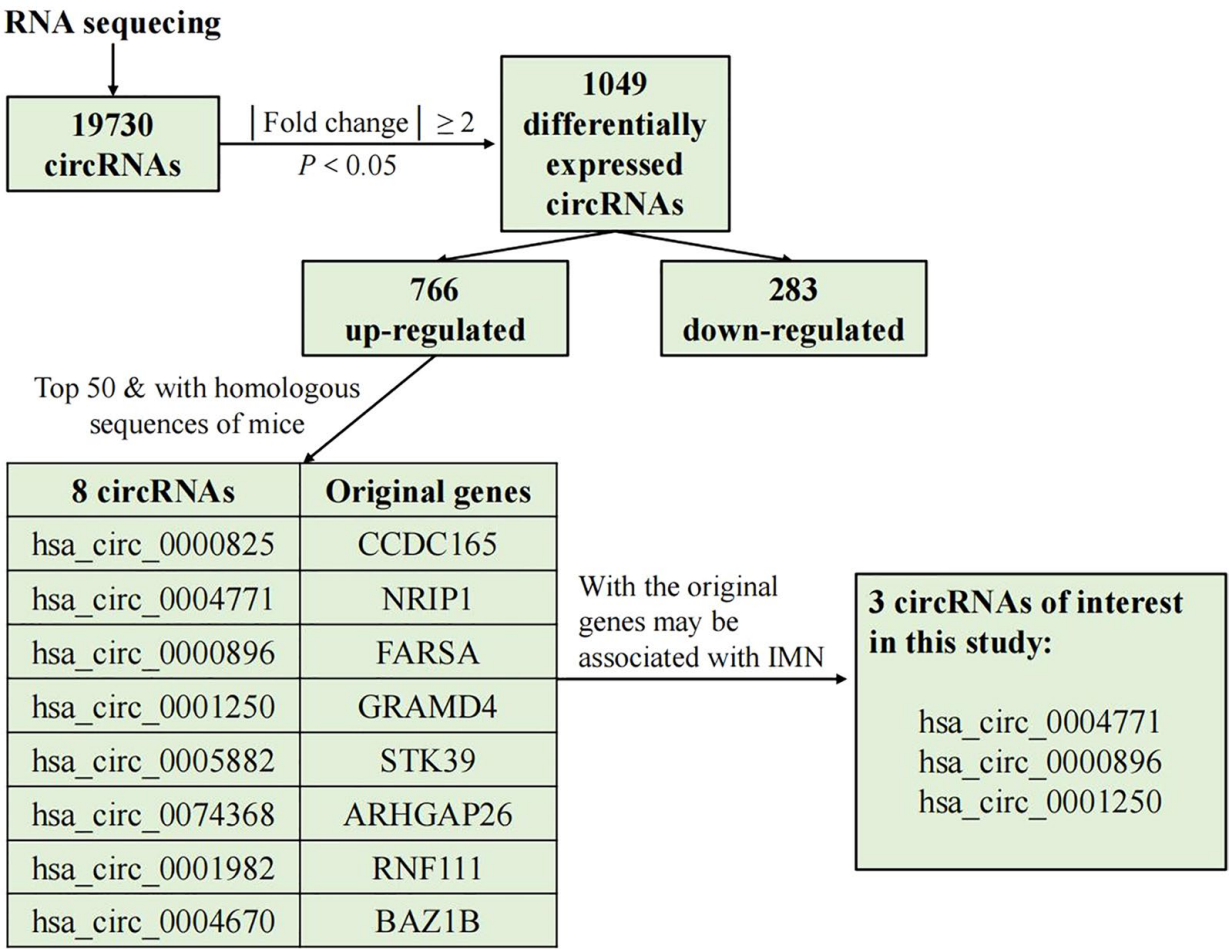
The main process of circular RNAs sequencing

Total RNA was reverse transcribed to synthesize cDNA using a Prime Script RT Reagent Kit (Perfect Real Time; TaKaRa, Osaka, Japan). Then quantitative polymerase chain reaction (qPCR) assay was conducted in triplicate with specific primers of different expressed circRNAs. To find biomarkers for IMN, we targeted the top 50 up-regulated circRNAs according to the value of log2 fold change of the sequencing results. As shown in Fig. 1, to carry on in vivo experiments on mice in the further study, we screened those circRNAs matched conserved mouse circRNAs with circBank database (http://www.circbank.cn/). Finally, we screened out the top 3 circRNAs, the original genes of which may be associated with renal diseases: hsa_circ_0004771, hsa_circ_0000896 and hsa_circ_0001250. The primers were chemically synthesized and validated in RiboBio Company (RiboBio, Guangdong, China), ACTB was used as an internal reference of exosomal circRNAs in urine. PCR primers are listed as follows: hsa_circ_0004771: F: CCGGATGACATCAGAGCTACT R: GTGCATCTTCTGGCTGTGTT hsa_circ_0000896: F: CACCGAGATGCCGACTGATA R: TGCTTGGAGATGCTGGTACT hsa_circ_0001250 F: GGTCACCTGGAAATTGCCTT R: CCGCTAGATCGAGGAAGTCA. ACTB F: AAGGTGACAGCAGTCGGTT R: TGTGTGGACTTGGGAGAGG. qPCR assay was conducted on ABI 7500 real-time PCR system (Applied Biosystems, CA, USA) used SYBR Green (Takara, China). All procedures were performed as follows: 95 °C, 10 min, 40 PCR cycles (95 °C, 15 s; 60 °C, 30 s, 72 °C, 20 s (fluorescence collection)). After the amplification reaction was finished, the procedure was performed as follows: 95 °C, 10 s; 60 °C, 60 s; and 95 °C, 15 s. Then the temperature was slowly increased from 60 to 99 °C (Automatic instrument, Ramp Rate 0.05 °C/s) to establish the melting curve. The relative expression levels of circRNAs were calculated by the 2^−ΔΔCq^ method.

### Detection of hsa_circ_0001250

#### Sanger sequencing

Sanger sequencing covering the backsplicing junction sequence was performed using the qPCR product of hsa_circ_0001250 with the process mentioned above. Divergent primers are used to amplify the full-length of hsa_circ_0001250, qRT-PCR assay was carried out using the following primers: F:GGTCACCTGGAAATTGCCTT R:TTTGATCTCATTCAGTCGAT. Sanger sequecing was performed by Sangon Biotech (Shanghai, China).

#### RNase R digestion assay

Total RNA was handled with RNase R (Geneseed Biotech Co., Ltd., Guangzhou, China) to confirm the circular structure of hsa_circ_0001250. Total RNA (2 μg) was incubated for 30 min at 37 °C with or without 3 U/μg RNase R. After RNase R treatment, PCR product agarose gel electrophoresis were performed to detect cDNA samples. PCR assay was performed using Easy-Load™ PCR Master Mix (GREEN, 2X). The PCR amplicons were visualized under a Molecular Imager®Gel Doc™ XR + System (Bio-Rad, USA) after electrophoresis in a 1.5% agarose gel electrophoresis with TAE buffer using a 100 bp DNA ladder (Takara, China).

### Statistical analysis

Statistical analysis was performed by SPSS 26.0 (Statistical Package for the Social Sciences, IBM Corp., Armonk, NY, USA) and GraphPad Prism (version 8.0.1 for Windows, GraphPad Software, San Diego, California USA, www.graphpad.com). Student's t-test was employed to analyse two-group comparisons. One-way ANOVA or Kruskal–Wallis multiple comparison test were used to compare measurement data among multiple groups. ROC curve analysis was performed to explore the diagnostic potential of hsa_circ_0001250. Pearson correlation analysis was used to analyze the relationship between the expression levels of hsa_circ_0001250 and the clinical features. Continuous variables with normal distributions are expressed as the mean ± standard deviation, otherwise are expressed as medians with interquartile ranges. A value of *P* < 0.05 was considered statistically significant.

## Results

### Clinical characteristics of the patients

The basic characteristics of 5 IMN patients and 5 HCs enrolled for RNA high throughput sequencing are summarized in Table 1. The basic characteristics of 23 IMN patients, 19 INS patients and 23 HCs enrolled in qPCR assay are listed in Table 2. There were no statistically significant differences among IMN, INS and HC groups with regarded to age, sex or BMI distribution (*P* > 0.05). The clinical characteristics including serum creatinine and eGFR didn’t show statistical differences (*P* > 0.05) while serum albumin, serum cholesterol and are significantly different between IMN and HC groups (*P* < 0.05).

**Table1:** Clinical characteristics of IMN and HC

Variables	Case (n = 5)	Control (n = 5)	*P*
Sex: Male (%)	3 (60%)	4 (80%)	0.490
Age	57.60 ± 12.46	42.80 ± 8.87	0.062
Serum creatinine (μmol/l)	81.07 ± 21.24	66.94 ± 11.69	0.229
eGFR (ml/min/1.73 m^2^)	85.99 ± 11.14	120.25 ± 28.32	0.051
Serum albumin (g/l)	21.94 ± 8.73	42.93 ± 4.03	0.003^**^
Total cholesterol (mmol/l)	10.37 ± 3.66	5.48 ± 0.49	0.039^*^
Proteinuria (g/24 h)	7.21 ± 4.65	–	–

*eGFR* Estimated glomerular filtration rate

*P < 0.05, **P < 0.01

**Table 2:** Clinical characteristics of IMN, INS and HC

Variables	IMN组 (n = 23)	INS组 (n = 19)	HC组 (n = 23)	*P*
Sex: Male(%)	16 (69.6%)	11 (57.9%)	17 (73.9%)	0.528
Age	46.39 ± 13.48	37.32 ± 18.11	46.35 ± 10.60	0.181
Serum creatinine(μmol/l)	81.03 ± 19.91	82.43 ± 26.25	72.99 ± 12.32	0.244
eGFR (ml/min/1.73 m^2^)	96.33 ± 27.27	94.97 ± 35.37	101.47 ± 21.92	0.567
Serum albumin (g/l)	23.74 ± 2.88^a^	24.87 ± 2.47^a^	43.90 ± 3.64^b^	< 0.001^***^
Total cholesterol(mmol/l)	8.27 ± 2.46^a^	7.30 ± 0.87^a^	5.13 ± 1.00^b^	< 0.001^***^
Proteinuria (g/24 h)	5.22 ± 1.35^a^	5.79 ± 1.41^a^	–	0.187

The same letters (a or b, respectively) indicate non-signifcant diference between groups based on Kruskal Wallis multiple comparison test

*P < 0.05, **P < 0.01, ***P < 0.001

### Characterization of urinary exosomes

**Fig. 2:**
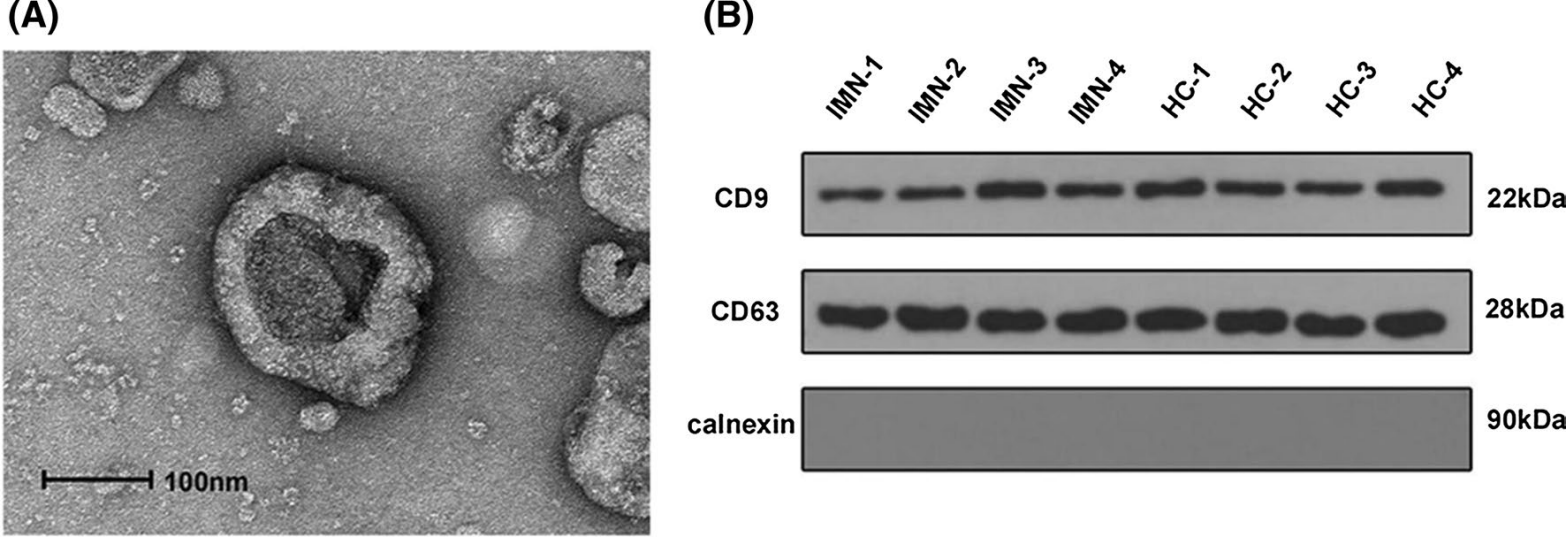
Detection of urinary exosomes. **A** The cup-shaped morphology with double-layer membrane structure of urinary exosomes was shown by TEM images. **B** Levels of specific exosomal markers as protein CD9, CD63, and calnexin as a negative control measured by Western blotting

As shown in Fig. 2, using a TEM, the characteristic cup-shaped morphology of the exosomes was observed with diameters about 40 ~ 160 nm (Fig. 2A). Exosomal protein markers including CD9, CD63 were detected by Western blot analysis. Results showed that CD9, CD63 were all highly enriched in the isolated exosomes, besides, calnexin as a negative control was not enriched (Fig. 2B).

### Profiling of differentially expressed urinary exosomal circRNAs of IMN and HC patients

**Fig. 3:**
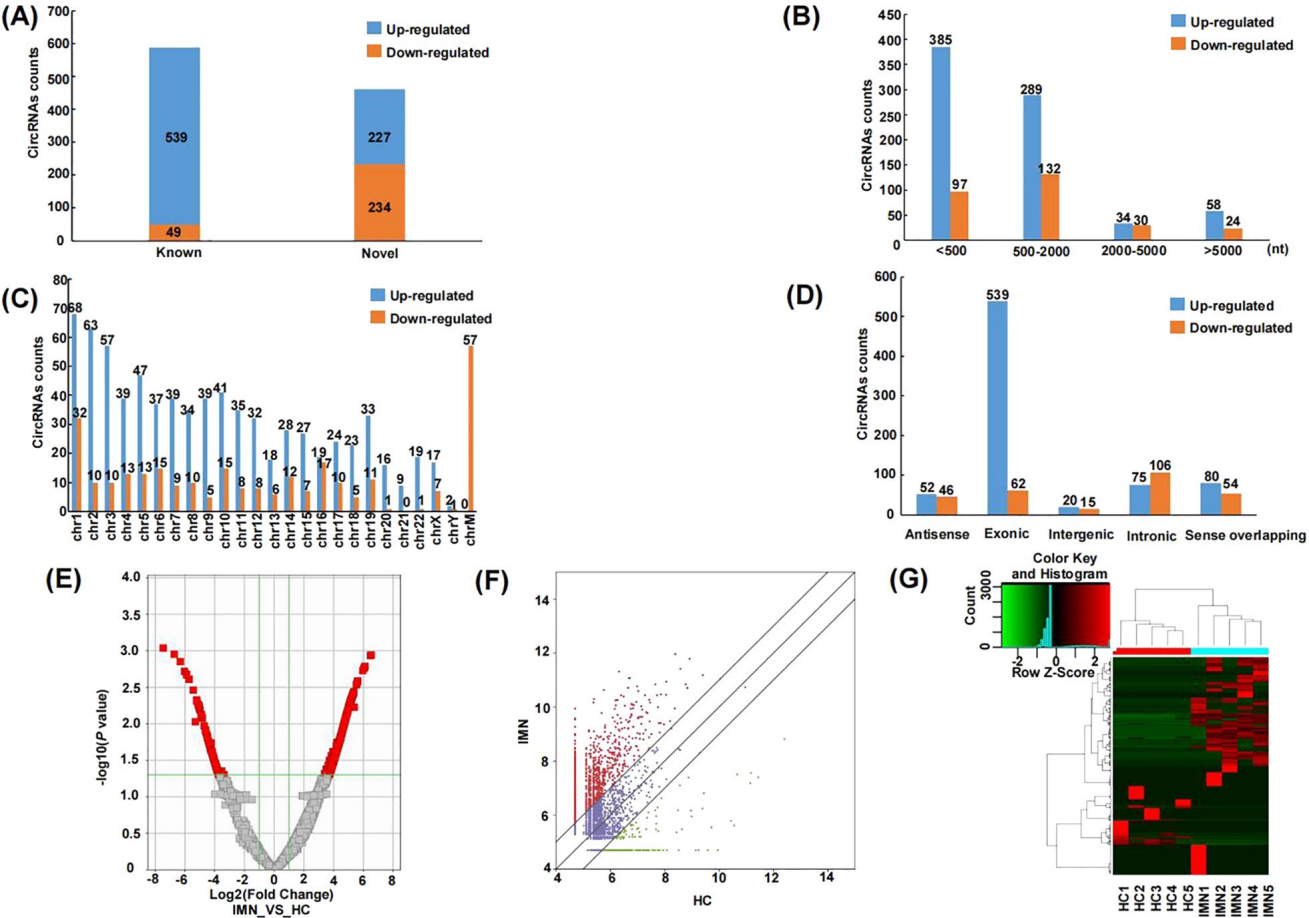
Profiling of differentially expressed urinary exosomal circRNAs of IMN and HC. **A** Percentage of novel and known differentially expressed circRNAs. **B** The counts of differentially expressed circRNAs based on their length. **C** The location of differentially expressed circRNAs on human chromosomes. **D** The distribution of differentially expressed circRNAs based on their categories. **E** Volcano plot of differentially expressed circRNAs between IMN and HC groups. Red dots represent circRNAs that are significantly differentially expressed. Outside the two vertical lines are the circRNAs with a Fold Change more than 2, the horizontal line indicates a *p*-value of 0.05. **F** Scatter plot of differentially expressed circRNAs in IMN and HC groups. Red and green dots indicate significant up-regulated and down-regulated circRNAs, respectively (with a fold change (FC) > 2.0 and *P* < 0.05). **G** Heatmap illustrating differentially expressed circRNAs between IMN and HC groups. The color key indicates the expression levels of circRNAs ranging from low (green) to high (red)

After RNA high throughput sequencing, a total of 19,730 transcripts of circRNAs were identified in all urinary exosome samples. As shown in Fig. 3, the results of the sequencing showed that there were 1049 differentially expressed circRNAs based on the threshold: fold change ≥ 2.0; *P* < 0.05. Compared with HC group, 766 transcripts were up regulated and 283 transcripts were down regulated in IMN group. Of the 1049 circRNAs, 461 were found for the first time and 588 have been found in previous studies (Fig. 3A). Most of the circRNAs are less than 2000 nt in length (Fig. 3B). These circRNAs were distributed on all chromosomes, including autosomal, sex chromosomes and some down regulated circRNAs were from mitochondrial genes (Fig. 3C). Among the 1049 circRNAs, exonic, intronic and other sources were accounted for 57.3% (601/1049), 17.3% (181/1049) and 25.4% (267/1049) respectively (Fig. 3D). Volcano plots (Fig. 3E) and scatter plot (Fig. 3F) displayed the differentially expressed circRNAs (FC ≥ 2.0; *P* < 0.05), most of which were up-regulated. Heat map and hierarchical clustering indicate that circRNAs were obviously different between IMN and HC groups (Fig. 3G).

### KEGG pathway analysis for the biological function of differentially-expressed circRNAs

**Fig. 4:**
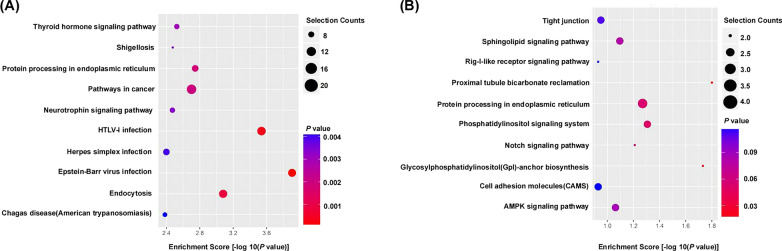
**A**, **B** Top 10 KEGG pathways with *P* values < 0.05 enriched for the up-regulated and down-regulated differentially expressed genes, respectively

We investigated the potential functions of differentially expressed circular RNAs using KEGG pathway analyse with the down-regulated and up-regulated differently expressed circRNAs, respectively. KEGG pathway analysis revealed that the up-regulated circRNAs participated in several key pathways such as Endocytosis and Protein processing in endoplasmic reticulum in the top 10 KEGG pathways enriched (Fig. 4A). Additionally, the down-regulated circRNAs were involved in Proximal tubule bicarbonate reclamation, Notch signaling pathway, Sphingolipid signaling pathway, AMPK signaling pathway, RIG-l-like receptor signaling pathway which may associated with pathogenesis of IMN (Fig. 4B).

### Validation of the high expression of hsa_circ_0001250 by qPCR

**Fig. 5:**
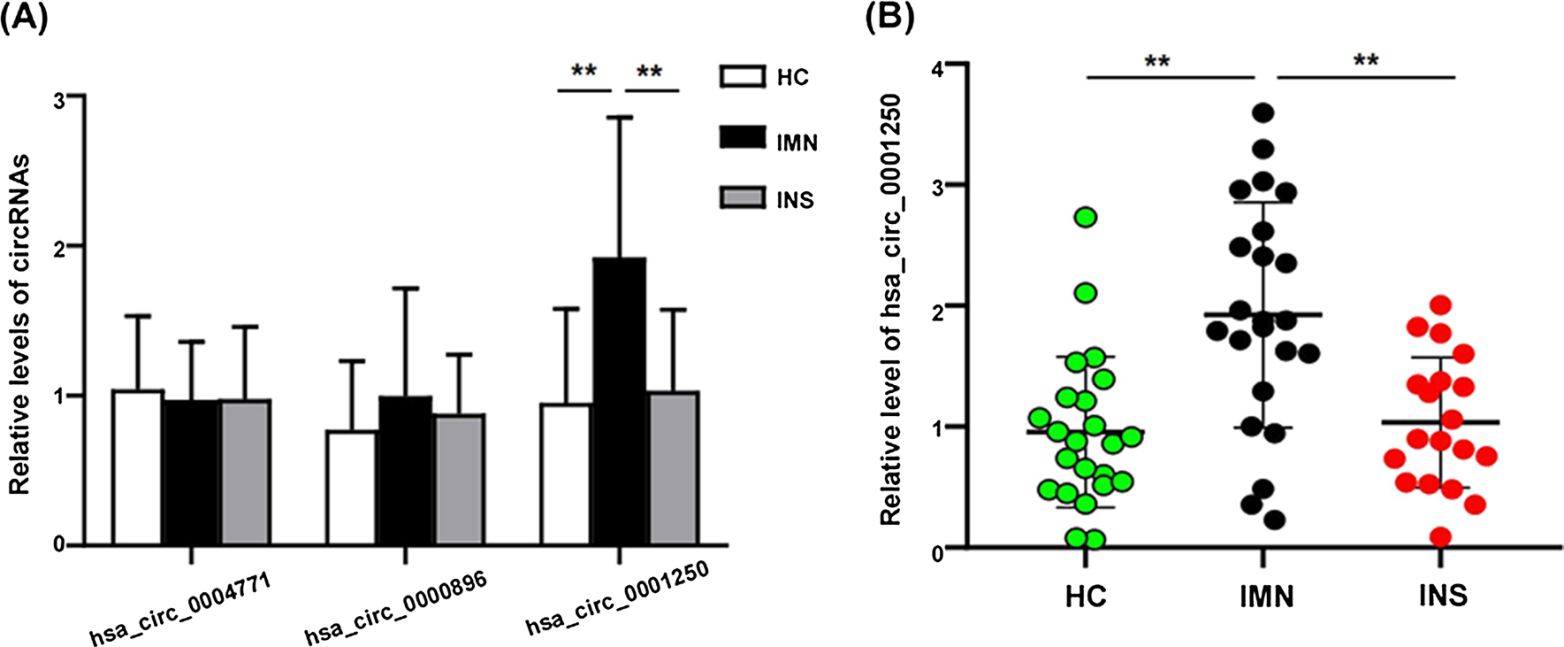
**A** Comparison of the expression level of following circRNAs between IMN, INS and HC groups by RT-qPCR: hsa_circ_0004771, hsa_circ_0000896 and hsa_circ_0001250, the expression of hsa_circ_0001250 was significantly increased in IMN. **B** Expression levels of hsa_circ_0001250 in urinary exosomes of IMN, HC and INS groups, showing the concrete distribution of hsa_circ_0001250 expression in the three groups. Data are presented as 2^−ΔΔCq^ relative to ACTB expression (mean ± standard deviation). **P < 0.01

To explore urinary exosomal circRNAs as biomarkers for IMN, we performed qRT-PCR assay among 23 IMN patients, 19 INS patients, and 23 HCs. The results of qRT-PCR showed that the expression of urinary exosomal hsa_circ_0001250 was significantly increased in IMN group than in the other two groups, while hsa_circ_0004771 and hsa_circ_0000896 didn’t show statistical difference among these 3 groups. This suggested that urinary exosomal hsa_circ_0001250 might be a novel potential diagnostic biomarker for IMN (Fig. 5).

### Detection of hsa_circ_0001250 by RNase R digestion and Sanger sequencing

**Fig. 6:**
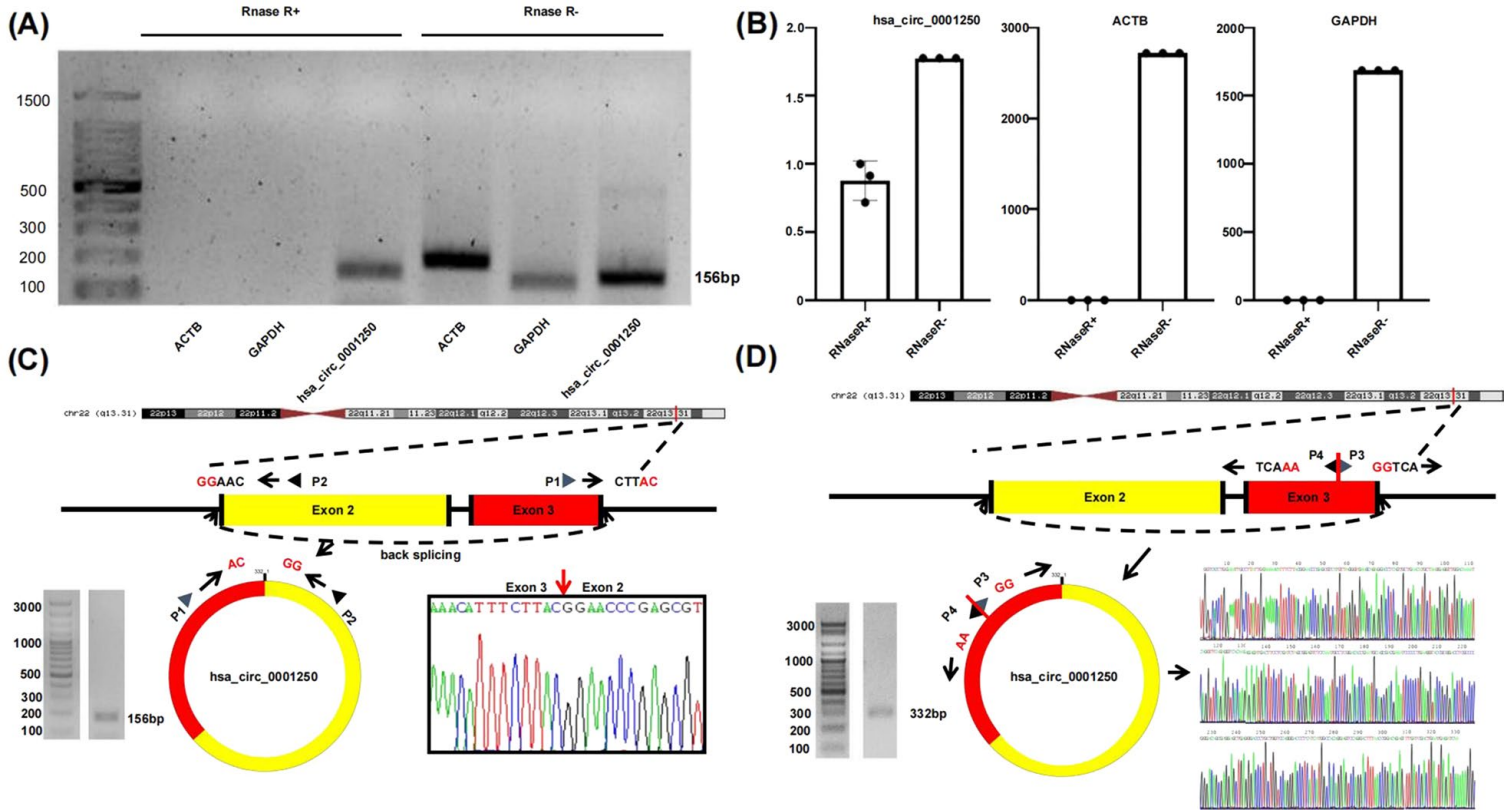
**A**, **B** PCR product agarose gel electrophoresis and qPCR indicated that the relative expression of ACTB and GAPDH in the RNase R + group was greatly reduced, while hsa_circ_0001250 was only slightly reduced. **C** Sanger sequencing for fragment contains the splicing junction further proved the ring structure of hsa_circ_0001250. **D** Sanger sequencing for full-length of hsa_circ_0001250 suggested that hsa_circ_0001250 was derived from exon 2 and exon 3 of the GRAMD4 gene and the sequence was consistent with data from circBase

The identified circRNAs of RNA sequencing were annotated with circBase database[30] and Circ2Traits[31]. After RNase R digestion, PCR product agarose gel electrophoresis and qPCR assay were performed to detect cDNA samples. Both of the results showed that the relative expression of linear ACTB and GAPDH in the RNase R + group was greatly reduced, while hsa_circ_0001250 was only slightly reduced (Fig. 6A, B). Compared with the linear ACTB and GAPDH, circRNA showed more tolerance, indicating that hsa_circ_0001250 is more resistant to digestion than linear RNA for the ring structure. These results of Sanger sequencing for fragment contains the splicing junction further proved the ring-structure of hsa_circ_0001250 (Fig. 6C). Sanger sequencing for full-length of hsa_circ_0001250 suggested that it was derived from exon 2 and exon 3 of the GRAMD4 gene, which was consistent with the hsa_circ_0001250 data from circBase. In addition, the sequence of the full length of hsa_circ_0001250 matched the data, as well (Fig. 6D).

### Diagnostic value of urinary exosomal hsa_circ_0001250 of IMN

**Fig. 7:**
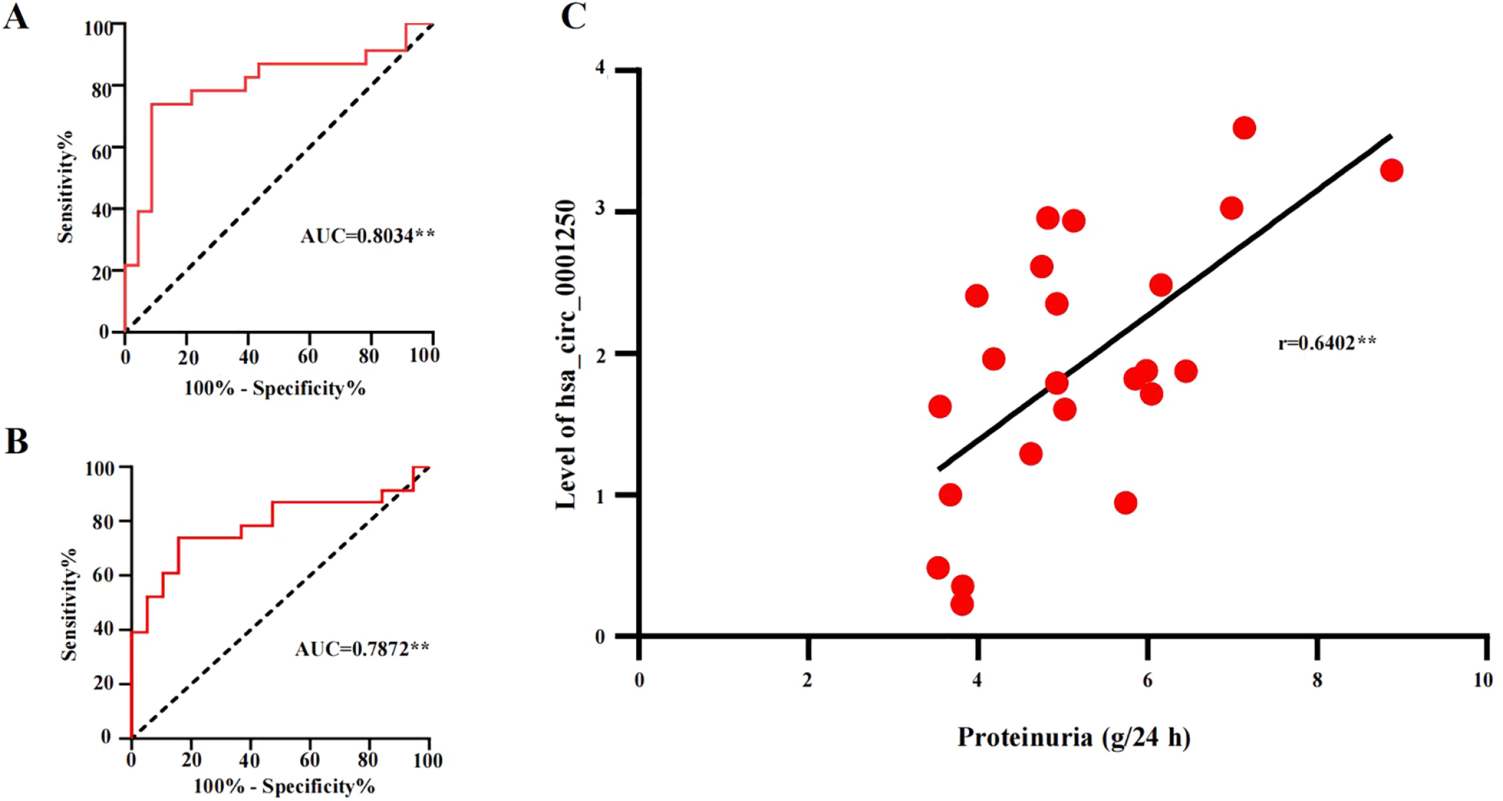
**A** ROC curves with a large AUC for hsa_circ_0001250 indicated its potential as a biomarker for IMN (AUC = 0.8034). AUC, area under roc curve; ROC, receiver-operating characteristic. **B** ROC curves showing the discriminatory ability of hsa_circ_0001250 to distinguish between IMN and INS (AUC = 0.7872). **C** Correlation Between hsa_circ_0001250 Expression and serum albumin level in patients with IMN. (r = 0.6402, **P < 0.01)

ROC curve analysis was used to further validate the potential utility of hsa_circ_0001250 as a diagnostic biomarker of IMN. As shown in Fig. 7, ROC curve analysis showed an association between hsa_circ_0001250 expression and idiopathic membranous nephropathy diagnosis. The area under the curve (AUC) to discriminate IMN from HC group was 0.8034 (95% confidence interval, 95%CI: 0.6667–0.9401) for hsa_circ_0001250, suggesting the high diagnostic potential of hsa_circ_0001250 in IMN patients (Fig. 7A). The AUC to discriminate IMN from INS group was 0.7872 (95% confidence interval, 95%CI: 0.6448–0.9296), suggesting hsa_circ_0001250 help differentiate between IMN and INS (Fig. 7B). Pearson's correlation coefficient indicated in the IMN group, high expression of hsa_circ_0001250 in IMN patients was significantly more relevant with high proteinuria level (r = 0.6402, *P* < 0.01) (Fig. 7C), while didn’t show obvious correlation with other clinical features (*P* > 0.05), as shown in Table 3.

**Table 3:** Correlation of the expression of the hsa_circ_0001250 and clinical features of IMN

Clinical characteristics	Correlation coefficient(r)	*P*
Age	0.100	0.649
Serum creatinine (μmol/l)	− 0.045	0.839
eGFR (mL/min/1.73 m2)	0.073	0.741
Serum albumin (g/l)	0.187	0.393
Serum cholesterol (mmol/l)	− 0.250	0.251
Proteinuria (g/24 h)	0.6402	0.001^**^

Pearson's correlation coefficient in IMN group, high expression of hsa_circ_0001250 in IMN patients was significantly relevant with high proteinuria level (r = 0.6402, **P < 0.01), was not relevant with other clinical features (*P* > 0.05)

### Prediction and annotation of hsa_circ_0001250 using a circRNA-miRNA-mRNA net work

**Fig. 8:**
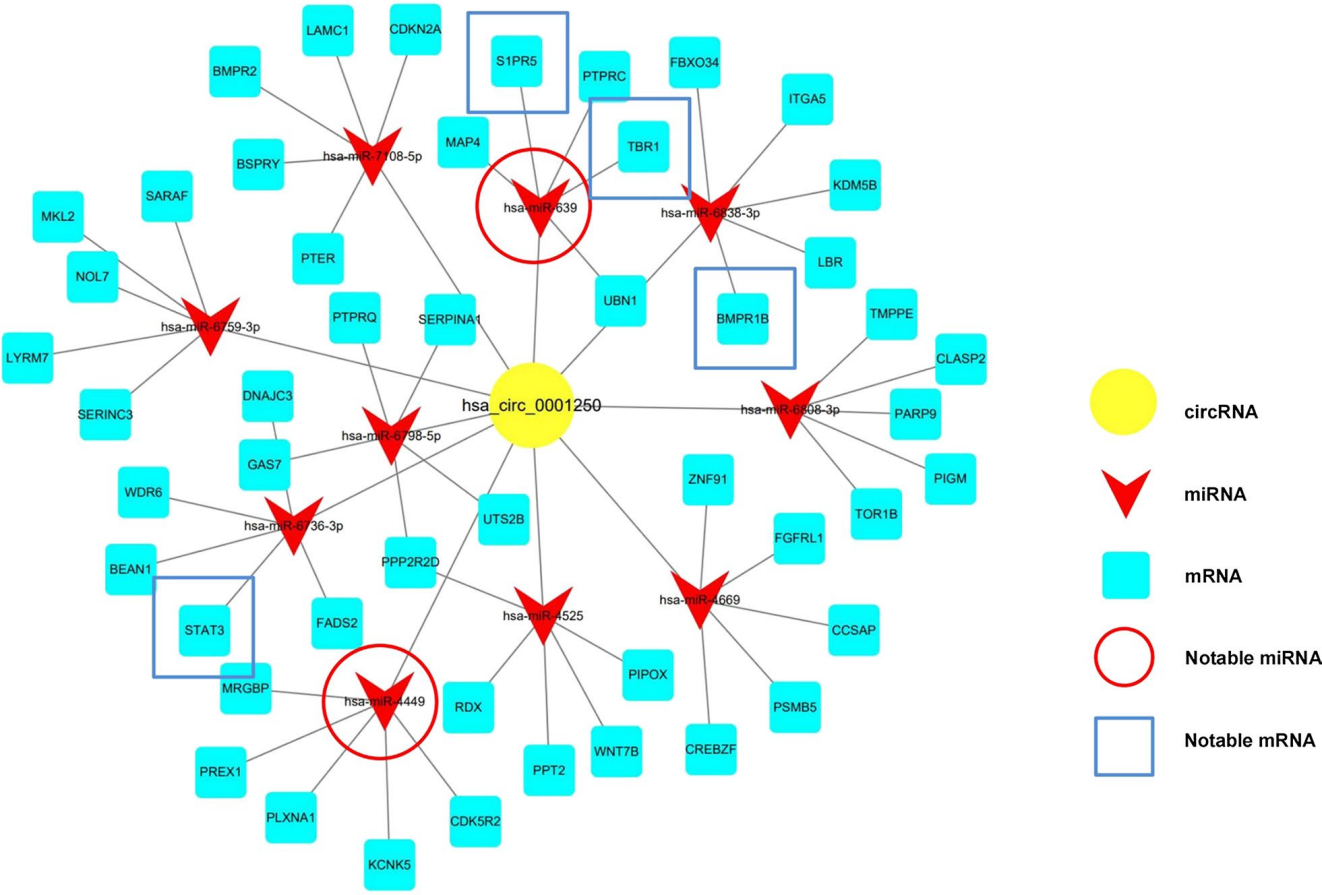
circRNA-miRNA-mRNA interaction network of hsa_circ_0001250. The association between the top 10 targeted miRNAs of hsa_circ_0001250 and their top 5 corresponding mRNAs. The red circles indicate miRNAs which may be more associated with IMN. The blue squares represent mRNAs which may participate the pathogenesis of IMN

To further determine the biological function and potential connections of hsa_circ_0001250, a circRNA-miRNA mRNA network was constructed. The top 10 miRNAs and top 5 mRNAs were theoretically predicted from hsa_circ_0001250(Fig. 8). We speculate that circRNAs may bind these miRNAs, after which the suppressive effect of miRNAs on the predicted target mRNAs would be abolished. Among the top ten corresponding miRNAs of hsa_circ_0001250, hsa-miR-639 and hsa-miR-4449 have been found to play a role in some pathogenesis, which may be involved in the pathogenesis and development of kidney disease. Among the predicted mRNAs, S1PR5, STAT3, TBR1, and BMPR1B were found to be related to some kidney diseases, as well. We have predicted downstream biological signaling in detail in the discussion part.

## Discussion

Various studies have focused on exosomal circRNAs for their stability and the function of transferring substances between cells. circRNAs derived from urinary exosomes contain pathophysiological information of a variety of kidney diseases, which can provide a diagnostic basis for kidney diseases [33, 34]. However, only a few researchers focus on the potentiality of exosomal circRNAs as diagnostic markers for idiopathic membranous nephropathy.

In this study, we used the second morning urine as sample material since it is less influenced by diet compared to the first morning urine [35]. What’s more, exosomes derived from the second morning urine were less affected by exosomes secreted by bladder epithelial cells and erythrocytes in some patients. At the same time, it also avoids the influence of changes in physical and chemical factors caused by staying in the bladder for too long. So we think second morning urine is most suitable for valid analysis of biomarkers in urine. We extracted total RNA from urinary exosomes and RNA high throughput sequencing analysis was performed between IMN group and HC group. We identified 1049 significantly differentially expressed circular RNAs (766 up-regulated and 283 down-regulated) in urinary exosomes RNA, among which 461 novel circRNAs were identified. The up-regulated circRNAs accounted for the majority of the known circRNAs, however, the down-regulated circRNAs account for half of all the novel circRNAs. The results suggested that there are many down-regulated circRNAs to be explored, and more functional experiments about them need to be carried out, as well. Additionally, these different expressed circRNAs were distributed on all chromosomes, interestingly, we found that most down-regulated circRNAs were derived from mitochondria. Studies have shown that mitochondria-derived circRNAs regulate the entry of proteins into mitochondrias and affect the function of mitochondrias [36]. Mitochondrial damage is closely related to kidney damage. Wang et al. have found that the traditional Chinese medicine Jianpi Qushi Recipe can delay renal pathological damage and inhibit the occurrence and development of membranous nephropathy by inhibiting cell apoptosis and up-regulating mitophagy through the PINK1/Parkin pathway [37]. Therefore, further researches on down-regulated urinary exosomal circRNAs in IMN could be focused on the above aspects. KEGG pathway analyse were used to explore the functions of these circRNAs. Results revealed they may participate in Endocytosis, Notch signaling pathway, Sphingolipid signaling pathway, AMPK signaling pathway, RIG-I-like receptor signaling pathway. Related researches could focus on these pathways.

Among differentially expressed circRNAs which may be involved in signaling pathways of pathogenesis of IMN and matched conserved mouse circRNAs, hsa_circ_0001250 was finally selected as the target circRNA, verified with qRT-PCR among IMN, INS and HC groups. To our knowledge, hsa_circ_0001250 is the first qPCR-tested urinary exomal circRNA after a RNA-squencing in IMN, the ring-structure of hsa_circ_0001250 was also confirmed in our research. What’s more, we text the results of RNA-sequecing by qPCR assay. Besides, a circRNA-miRNA-mRNA network is predicted for further understanding of functions of hsa_circ_0001250, leading to a more convincing conclusion.

The circRNAs identified through next generation sequencing and bioinformatics analysis needs to be further verified through experiments for their existence and biological functions. With the gradual deepening of circRNA research, the sequence of circRNAs were found occasionally different from original genes [38], so the identification and analysis of the full-length circRNA sequence becomes more and more important. Sanger sequencing for the full-length of hsa_circ_0001250 were performed to confirm the sequence of hsa_circ_0001250. Sanger sequencing for the fragment contains the splicing junction of hsa_circ_0001250 and RNase R digestion assay followed by PCR product agarose gel electrophoresis and qRT-PCR assay were carried to confirm the ring-structure of hsa_circ_0001250, which are necessary for further research.

Moreover, through accuracy prediction estimation (ROC curve analysis), urinary exosomal hsa_circ_0001250 was tested to serve as a diagnostic biomarker to predict IMN. At the same time, we were able to differentiate between INS and IMN, further supporting that the up-regulated expression of hsa_circ_0001250 are specific to IMN. Pearson's correlation coefficient showed that the expression of hsa_circ_0001250 in IMN patients is positively correlated with the level of proteinuria. We know that high level of proteinuria means progression of IMN, so we speculate that hsa_circ_0001250 may promote the progression of IMN, which requires further experimental verification. However, we didn’t find the relevance between hsa_circ_0001250 and other clinical features, so more samples are required in future experiments to verify our conclusion.

Previous studies speculated that exon-derived circRNAs may perform regulatory functions through a circRNA-miRNA-mRNA network in the cytoplasm, while intron-derived circRNAs seem to play regulatory roles in the nucleus [39]. Through RNA sequencing, we confirmed that hsa_circ_0001250 was derived from exon2 and exon3 of GRAMD4, so it was hypothesized that hsa_circ_0001250 may also regulate a circRNA-miRNA-mRNA network through a competitive endogenous RNA mechanism. A gene network is a group of genes that operate in a coordinated manner to control common functions [40], which mainly lies in gene-to-gene regulation, while circRNA-miRNA-mRNA is a network reflecting the regulation between non-coding RNAs and their target genes. At the molecular level, the sequences of circRNAs contain a large number of miRNA binding sites. When miRNAs were binded with circRNAs,the suppressive effect of miRNAs on target mRNAs would be abolished, resulting in a negative effect. These RNA transcripts are called endogenous competing RNAs (ceRNAs), and this new model of post-transcriptional regulation is called the ceRNA hypothesis [41]. In our research, a circRNA–miRNA–mRNA network was constructed to further understand the potential biological functions of hsa_circ_0001250. In the circRNA-miRNA-mRNA network, we can see among the top 10 miRNAs which may be regulated by hsa_circ_0001250, hsa-miR-639 was found associated with cell proliferation and cell cycle by targeting CDKN1A [42], TGFβ-induced EMT by targeting FOXC1 [43], proliferation and migration through the KAT7/Wnt/β-Catenin Pathway [44] in other diseases. In addition, hsa-miR-4449 was found regulating IL-1β and IL-18 expressions, the level of ROS, and pyroptosis in DKD pathogenesis [45]. So we supposed that hsa_circ_0001250 may also play a role in the pathogenesis IMN by target these miRNAs, which need to be proved in further research. Moreover, among the predicted targeted mRNAs, S1PR5, STAT3, TBR1 and BMPR1B were found to be related to some kidney diseases. The expression of S1PR5 was found up-regulated by TGF-β2 in a time- and concentration-dependent manner, which is a necessary condition for TGF-β to induce profibrotic of CTGF. S1PR5 may be a therapeutic target to treat renal fibrosis [46]. S1PR5 has also been found to be involved in the regulation of immune response [47]. In addition, STAT3 phosphorylation was found reduced by the overexpressed of circ_0007059, protecting cell viability and reducing inflammation in a nephritis cell model [48]. Moreover, Methylation of TBR1 is associated with the progression of renal cell carcinoma [49]. BMPR1B was up-regulated by the loss of oncogenic miR-1274a, reducing cancer cell proliferation and inducing apoptosis in clear cell renal cell carcinoma [50]. Therefore, further study on the mechanistic studies on hsa_circ_0001250 could be focused on these mRNAs. In addition, we didn’t find relevant articles confirming that these miRNAs or mRNAs are decreased or increased in IMN, therefore, follow-up experiments are needed to verify the circRNA-miRNA mRNA network.

However, as urinary exosomes may originate from a variety of cells, such as juxtaglomerular cells, podocytes, parietal cells, proximal tubular epithelial cells, and so on [51], the specific cell source of hsa_circ_0001250 remains unknown, we speculate that in IMN patients, the release of hsa_circ_0001250 may be caused by the damage of podocytes, which needs further experimental confirmation, as well.

## Conclusion

In conclusion, we identify a series of differentially expressed circRNAs in urinary exosomes collected from IMN patients and HCs. The identified circRNAs are involved in the biological processes associated with IMN. Moreover, we propose urinary exosomal hsa_circ_0001250 to be a potential candidate biomarker of IMN and predict a potential circRNA-miRNA-mRNA network. To our knowledge, this is the first time the correlation between hsa_circ_0001250 and diseases that has been studied. These findings may point out the direction for further research on molecular pathogenesis of IMN.

## Data Availability

Raw reads of the sequencing project have been successfully deposited to SRA database with BioProject ID: PRJNA793462.

## Abbreviations

IMN: Idiopathic membranous nephropathy
INS: Idiopathic nephrotic syndrome
HC: Health control
qPCR: Quantitative polymerase chain reaction
ROC: Receiver operating characteristic
KEGG: Kyoto Encyclopedia of Genes and Genomes
circRNA: Circular RNA
MN: Membranous nephropathy
TEM: Transmission Electron Microscopy
WB: Western blot
ROC: Receiver operating characteristic
BMI: Body Mass Index
eGFR: Estimated Glomerular Filtration Rate
BP: Biological process
